# Survival of the Stillest: Predator Avoidance in Shark Embryos

**DOI:** 10.1371/journal.pone.0052551

**Published:** 2013-01-09

**Authors:** Ryan M. Kempster, Nathan S. Hart, Shaun P. Collin

**Affiliations:** The Oceans Institute and the School of Animal Biology, The University of Western Australia, Crawley, Western Australia, Australia; University of Lethbridge, Canada

## Abstract

Sharks use highly sensitive electroreceptors to detect the electric fields emitted by potential prey. However, it is not known whether prey animals are able to modulate their own bioelectrical signals to reduce predation risk. Here, we show that some shark (*Chiloscyllium punctatum*) embryos can detect predator-mimicking electric fields and respond by ceasing their respiratory gill movements. Despite being confined to the small space within the egg case, where they are vulnerable to predators, embryonic sharks are able to recognise dangerous stimuli and react with an innate avoidance response. Knowledge of such behaviours, may inform the development of effective shark repellents.

## Introduction

Electroreception is found throughout the animal kingdom from invertebrates to mammals and has been shown to play an important role in detecting and locating prey [1], [2], mates [3], potential predators [4], [5] and is thought to be important in orienting to the earth's magnetic field for navigation [6]–[8]. Electroreceptors of sharks, the ampullae of Lorenzini, detect minute electric field gradients via an array of openings or ‘pores’ at the skin's surface [2]. Spatial information on the location of a field source is assessed by the differential stimulation of ampullae as the position of the source changes relative to the animal [1], [2], [6], [9]. The spatial separation and arrangement of each pore in the array directly influences the detection of electric stimuli and the resultant changes in the shark's behaviour [2], [10].

**Figure 1:**
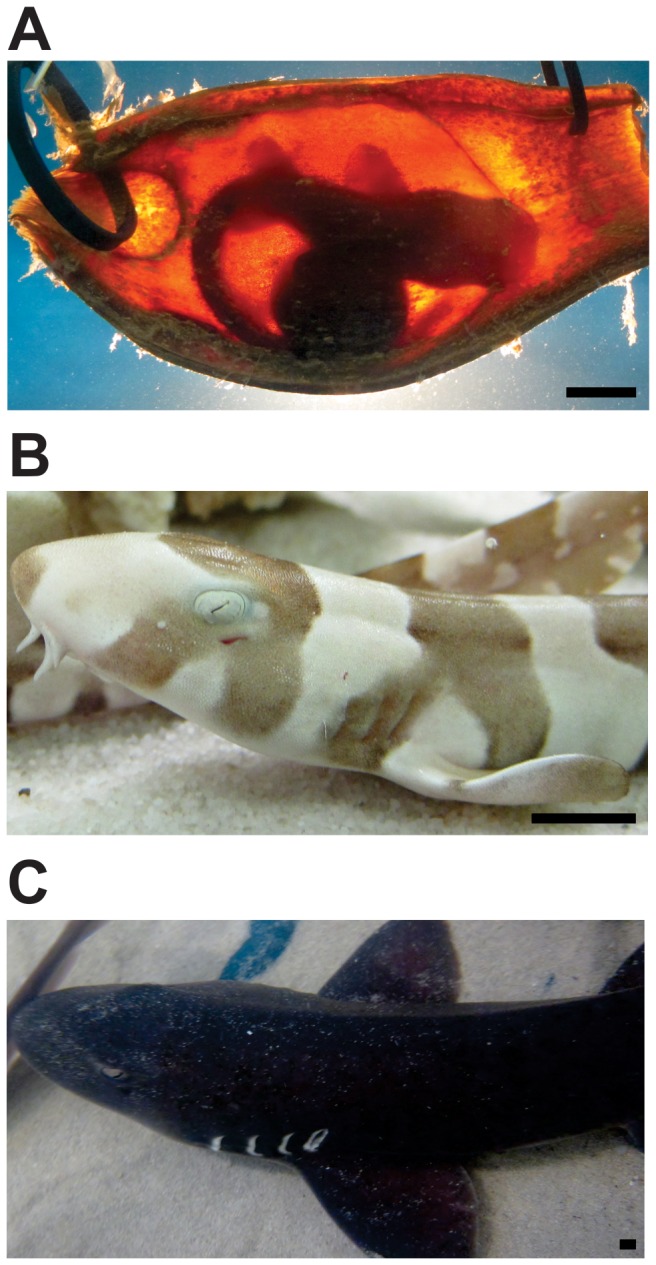
A–C. Photographs depicting three major life stages of the bamboo shark (*Chiloscyllium punctatum*). **A**: Embryo encapsulated within an egg case. **B**: Early juvenile (post hatching) showing its high contrast banding pattern. **C**. Sexually mature individual that has lost its banding leaving a more typical counter-shading pattern, which it uses to camouflage itself on the substrate. Scale bars = 10 mm.

The electrosensory system of adult sharks is known to primarily mediate the passive detection of bioelectric stimuli produced by potential prey [1], [2]. However, it has been postulated that the electroreceptive system can be used to detect, and thus avoid, potential predators [4]. Shark embryos that develop inside their mother may have little or no use for electroreception until birth, given that they are protected within the uterus and are nourished either directly by their mother (viviparity) or via an external yolk sac (ovoviviparity). However, oviposited embryos like those of the bamboo shark (*Chiloscyllium punctatum*) develop completely independently of their mother inside an egg case (oviparity) (Fig. 1A) [11]. These egg cases are typically deposited on or near the substrate, where they are vulnerable to predators including other sharks, teleost fishes, marine mammals and even large molluscan gastropods [12], [13].

*Chiloscyllium punctatum* embryos will spend up to five months encapsulated inside a leathery egg case without the opportunity to escape or visually detect the approach of predators (Fig. 1A) [11]. After hatching, at just 10–12 cm in length [11], bamboo shark juveniles are extremely vulnerable to predation. However, at this stage, their distinctive pattern of high contrast banding (Fig. 1B) may assist in avoiding predators since these conspicuous bands mimic the colouration of unpalatable or poisonous prey, *i.e.* sea snakes, thereby avoiding predation (known as Batesian mimicry). This potentially aposematic colouration is lost as the bamboo shark reaches maturity and the banded pattern fades. As it matures, this species adopts the more familiar counter-shading pigmentation exhibited by many other species of sharks, thereby enabling it to camouflage itself against a dark substrate (Fig. 1C).

During early embryonic development (stages 3–25) [11], [14], bamboo sharks are sealed within a pigmented egg case, where their presence would be masked to any visually-driven predators and there would be no exchange of fluids [11] with the surrounding seawater, negating their detection via either mechanoreceptive (lateral line) or olfactory signals. However, as the embryo approaches the pre-hatching stage of development (stages 26–32), the bottom edge of the egg case weakens and the marginal seals open, thereby allowing the entry of seawater [11] and the release of sensory cues that may be detectable by predators. As the embryo increases in size, it begins to undulate the tail to facilitate circulation of fresh seawater through the open seals of the egg case to assist in respiration. However, this is thought to increase the risk of predation [13] owing to the greater likelihood that a passing predator could detect the presence of the embryo due to the release of olfactory cues and/or intermittent hydrodynamic disturbances. Following an increase in the frequency of tail undulations and respiratory gill movements, between stages 26 and 32, the electrosensory system differentiates and may become functional by stage 32 [15], presumably to assist in predator detection prior to hatching [4].

## Results and Discussion

**Figure 2:**
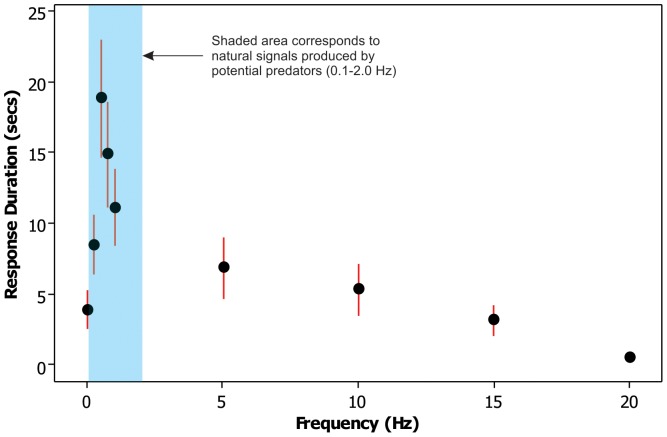
Average freeze response duration (±2×Standard Error) of bamboo shark embryos (stage 32–34) to a range of sinusoidal frequencies (0–20 Hz) and stimulus intensities (0.4–2.1 µV/cm). Shaded bar corresponds to natural respiratory signals produced by potential predators (1.0–2.0 Hz) [22] and low frequency modulations of D.C. fields produced by approaching predators as they move relative to an object (0.1–1.0 Hz) [23]. Peak response frequency: 0.5 Hz; duration: mean 18.9 secs.

When exposed to predator-simulating sinusoidal electric fields, late stage bamboo shark embryos (stage 34) respond by the cessation of all respiratory gill movements, thereby minimising their own electrosensory and mechanosensory output in order to avoid detection (Fig. 2). The cessation of gill movements is immediately followed by a rapid coiling of the tail around the body, with little or no discernible body movement during exposure (‘freeze’ response). Vertebrates that exhibit a ‘freeze’ response to predators have also been shown to induce cardioventilatory responses, where they decrease their heart rate (bradycardia) to reduce predation risk [16]–[21]. As a result, the length of time that an animal is able to respond is finite, as the need to breathe and pump oxygen around the body will eventually overcome the urge to remain still and undetected. Thus, the bamboo shark embryos tested eventually resume, albeit much reduced, gill movements whilst still being exposed to the predator-simulating stimuli.

**Figure 3:**
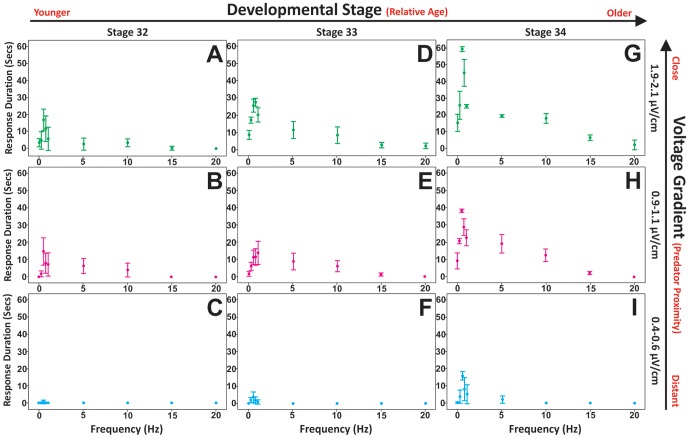
A–I. Freeze response duration (±2×Standard Error) of bamboo shark embryos (stages 32–34) to a range of sinusoidal frequencies (0–20 Hz) and stimulus intensities (0.4–2.1 µV/cm). Embryos are categorised into nine groups according to their relative stage in development and intensity of the electric field strength exposure. **A**: Stage 32 embryos exposed to 1.9–2.1 µV/cm (peak response frequency: 0.5 Hz; duration: mean 16.7 secs). **B**: Stage 32 embryos exposed to 0.9–1.1 µV/cm (peak response frequency: 0.5 Hz; duration: mean 14.9 secs). **C**: Stage 32 embryos exposed to 0.4–0.6 µV/cm (peak response frequency: 0.5 Hz; duration: mean 0.3 secs). **D**: Stage 33 embryos exposed to 1.9–2.1 µV/cm (peak response frequency: 0.75 Hz; duration: mean 27.7 secs). **E**: Stage 33 embryos exposed to 0.9–1.1 µV/cm (peak response frequency: 1.0 Hz; duration: mean 13.8 secs). **F**: Stage 33 embryos exposed to 0.4–0.6 µV/cm (peak response frequency: 0.5 Hz; duration: mean 3.7 secs). **G**: Stage 34 embryos exposed to 1.9–2.1 µV/cm (peak response frequency: 0.5 Hz; duration: mean 59.4 secs). **H**: Stage 34 embryos exposed to 0.9–1.1 µV/cm (peak response frequency: 0.5 Hz; duration: mean 38.4 secs). **I**: Stage 34 embryos exposed to 0.4–0.6 µV/cm (peak response frequency: 0.5 Hz; duration: mean 15.8 secs).

Bamboo shark embryos (stage 32–34) show the greatest avoidance response to sinusoidal electric field frequencies between 0.25 and 1.00 Hz (peaking at 0.5 Hz; Fig. 2), with response duration (measured from initial time of exposure) increasing as the electric field strength increases (increasing electric field strength may simulate closer and/or larger predators) (Fig. 3). Less developed embryos (stages 32–33) exhibit a reduced response duration to predator-simulating stimuli (Fig. 3A–F). Embryos as young as stage 32 would only respond if the electric field was of sufficient strength, approximately ≥0.9 µV/cm (Fig. 3B). In contrast, stage 34 embryos would respond to electric field strengths as low as 0.4 µV/cm (Fig. 3I). Embryos prior to stage 32 failed to show any response to electric field strengths between 0.4 µV/cm and 2.1 µV/cm.

**Figure 4:**
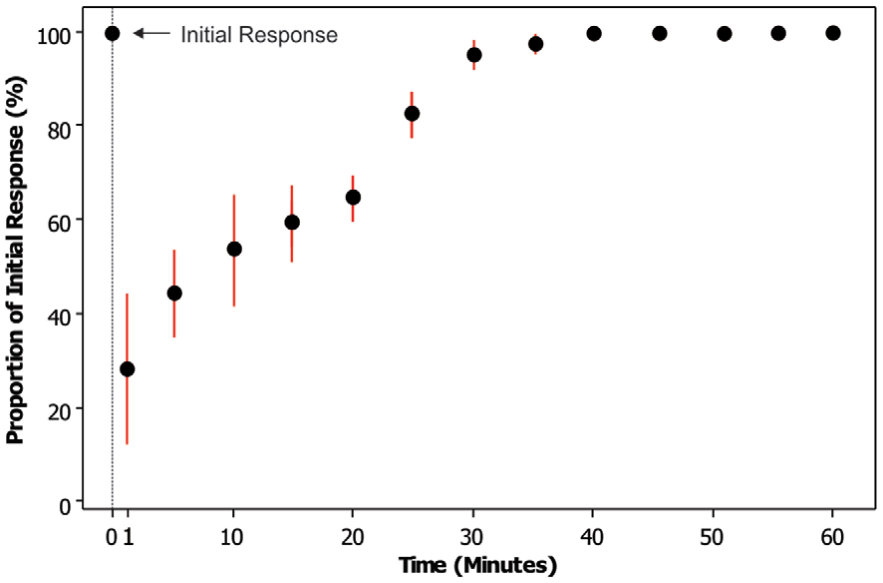
Relative freeze response duration when embryos (stage 34) are repeatedly exposed to the same stimulus at set time intervals after the first (initial) response. Embryos were individually exposed to the same stimulus to get an average initial response time. Embryos were then exposed to the same stimulus 60 minutes after initial response, and re-exposed at decreasing time intervals. Response duration is expressed as a percentage of the initial response.

These results agree with the differentiation and development of the electrosensory system, as has been previously shown for the lesser spotted catshark (*Scyliorhinus canicula*) when the ampullary organs become innervated [5], [15]. Repeated exposure to the same stimulus also resulted in a reduced response duration as embryos (stages 34) became desensitised; embryos appeared to recognise previously presented stimuli when repeatedly exposed within a 30–40 minute period (Fig. 4). In contrast, the lesser spotted catshark and the clearnose skate (*Raja eglanteria*) habituate to stimuli within only 5 to 10 minutes of the initial exposure, respectively [4], [5], highlighting significant species-specific differences in the level of temporal sensitivity of the electrosensory system in elasmobranchs.

The greatest avoidance response to sinusoidal electric fields (0.25–1.00 Hz with a peak at 0.5 Hz; Fig. 2) exhibited by bamboo shark embryos in this study corresponds to the natural respiratory signals produced by their potential predators, *i.e.* teleosts and other elasmobranchs [3], [4], [13], [22], and the low frequency modulations of D.C. fields produced by approaching predators [23]; thus indicating the important function of electroreception in the detection and avoidance of predators.

This study advances our understanding of how embryonic sharks respond to electric fields of specific frequency and intensity and how their survival instincts to feed and defend themselves may take precedence over an electrical deterrent under some conditions [24]. The conditions under which this species habituates to electrical stimulation may also be useful in the development of electrical shark repellent devices.

## Materials and Methods

### Ethics Statement

This study was carried out in strict accordance with the guidelines of the *Australian Code of Practice for the Care and Use of Animals for Scientific Purposes* (7^th^ Edition 2004) *‘The Code’*. The protocol was approved by the University of Western Australia Animal Ethics Committee (Permit No. RA/3/100/917). Embryos were monitored daily to assess activity levels before, during and post stimulation to allow adequate rest time between experimental trials, and all efforts were made to minimise suffering.

### Collection and staging of embryos

Bamboo shark embryos were collected as freshly oviposited egg cases from captive bred adults from Underwater World and Daydream Island Resort aquaria in Queensland, Australia. To enable video recording of embryo activity within the egg case, the opaque external fibrous layer of each egg case was scraped off upon collection. Developing embryos could then be seen clearly through the transparent inner layer when held in front of a fibre optic light source. Eggs remained submerged in a shallow petri dish filled with seawater throughout this procedure. The embryos were monitored for developmental changes and compared to the stages described for *Chiloscyllium punctatum* [11] and the staging criteria outlined for *Scyliorhinus canicula* [14]. All stages, ranging from when the embryo could first be observed with the unaided eye (stage 14), through to pre-hatching, fully developed embryos (stage 34), were tested. Developmental changes were only recorded in the most advanced embryos (stages 31–34).

### Experimental design

**Figure 5:**
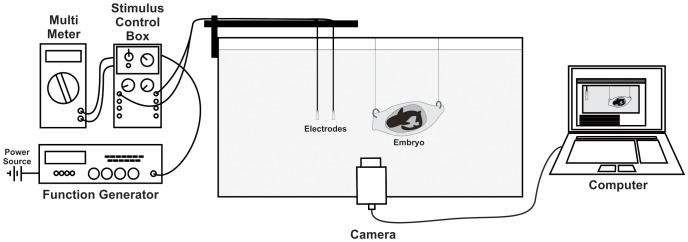
Experimental apparatus used to study the response of bamboo shark embryos to predator simulating dipole electric fields. A function generator and stimulus controller were used to deliver a dipole electric field of specific intensity and frequency to electrodes positioned along the same longitudinal axis as the embryo. Embryo responses were recorded with a video camera positioned directly in front of the experimental tank.

Embryos encapsulated within the egg case were suspended in a 90 cm long, 45 cm wide, 50 cm deep glass aquarium and transferred individually to an identical tank for testing. A total of 11 embryos were stimulated with sinusoidal electric fields (0–20 Hz) at various stages in their development (stages ≤31–34). Electric stimuli were applied at three major intensities (0.4–0.6 µV/cm, 0.9–1.1 µV/cm and 1.9–2.1 µV/cm) via a function generator and a custom built stimulus generator [25] with an applied current between 100 µA and 500 µA (Fig. 5). To ensure minimal variation in the electric field produced, water temperature of 24–25°C and water resistivity of 18–19 Ω cm were maintained.

To account for non-responses, embryos were each stimulated the same number of times (3 replicates of each stimulus strength and frequency combination: 27 tests in total) and all test results (including zeros) were used to determine the average response duration. Therefore, each embryo was stimulated a total of 27 times (per developmental stage) to obtain a full data set covering all frequency variations (0–20 Hz) and all stimulus strength variations (0.4–2.1 µV/cm).

Electric stimuli intensity (i.e. voltage gradient, V/cm) at the position where the embryo responds to the stimulus was calculated using the equation, *V/cm = (ρ.I.d. cosα)/(2πr^3^)* [26], based on the ‘ideal dipole field’ equation [27] and the ‘charge distribution of an electric field’ equation [28]. The variables are as follows: *ρ* is the resistivity of the seawater (Ω cm), *I* is the applied electric current (A), *d* is the distance between the two electrodes of a dipole (cm), r is the radius (the distance from the centre of the dipole to the position in space where the potential is being calculated) and *α* is the angle from the position in space to the centre of the dipole with respect to the axis.

In pre-experimental trials, shark embryos appeared to show an increased response duration when the electrode separation distance was increased, indicating that the embryos may interpret this as an increase in the size of the simulated predator [28], [29]. To reduce these experimental variables, the electrode separation distance was set at 5 cm with the embryo held at a uniform radius of 12 cm from the dipole source. These measurements were based on tank size restrictions to minimise any backscatter effects. Further investigation is encouraged to better understand the effect of increasing electrode separation distance on predator avoidance response (repellent effect).

The stimulus generator enabled the strength of the applied current to be varied and the function generator enabled a specific wave form to be selected and the output frequency controlled. An ammeter in series allowed the amount of current being applied through the circuit to be monitored in order to establish that the circuit was complete, thereby confirming that an electric field was being generated between the electrodes in the tank. Current from the stimulus generator was delivered to the electrodes via submerged cables and seawater-filled polyethylene tube salt bridges. A pair of shielded 18AWG coaxial underwater cables was plugged into the stimulus generator. Current was passed to seawater-filled polyethylene tubes via the exposed stainless steel pins of the cables [1]. The seawater-filled polyethylene tubing formed a salt bridge between the electrode arrays in order to eliminate eddy currents due to inhomogeneities on the electrode surface [4]. The electrodes were positioned adjacent to the egg case along the longitudinal axis (Fig. 5). During the behavioural observations, stimulus frequencies were presented as continuous sinusoidal stimuli. Response to the stimulus was determined by cessation of all gill movements or a ‘freeze’ response [Movie S1]. Embryos were stimulated for a minimum of 10 seconds after they resumed initial gill movements, to ensure that the resumption of breathing had been accurately identified. In order to avoid habituation to the electrical stimulus, an inter-trial interval of 40 minutes was used after each freeze response was observed. The strength and frequency of the stimuli was also varied pseudorandomly.

### Video analysis

All behavioural trials were recorded in high definition using a Canon S95 digital video camera. The camera was positioned to view the embryo and at least one of the electrodes (Fig. 5). As the electrodes were positioned along the same longitudinal axis as the embryo they could later be used as a calibration for measurements taken directly from the video clips (the electrodes were a known diameter) including embryo and yolk size and also to confirm embryo distance from the electrode source. Audio from the video was used to determine the point at which the stimulus source was switched on and off. The video analysis software Kinovea™ was used to assess behavioural clips and record response time.

## Supporting Information

Movie S1 **Video clip of a bamboo shark embryo (stage 33) responding to an electrical stimulus (stimulus strength: 0.25 Hz; 0.9–1.1 µV/cm) by ceasing gill movements.**
(MPG)

## References

[1] Kalmijn A (1971) The electric sense of sharks and rays. J Exp Biol 55: 371–383. 5114029 10.1242/jeb.55.2.371

[2] Kempster RM, McCarthy ID, Collin SP (2012) Phylogenetic and ecological factors influencing the number and distribution of electroreceptors in elasmobranchs. J Fish Biol 80: 2055–2088. 22497416 10.1111/j.1095-8649.2011.03214.x

[3] Tricas TC, Michael SW, Sisneros JA (1995) Electrosensory optimization to conspecific phasic signals for mating. Neurosci Lett 202: 129–132. 8787848 10.1016/0304-3940(95)12230-3

[4] Sisneros JA, Tricas TC, Luer CA (1998) Response properties and biological function of the skate electrosensory system during ontogeny. J Comp Physiol A 183: 87–99. 9691481 10.1007/s003590050237

[5] Peters RC, Evers HP (1985) Frequency selectivity in the ampullary system of an elasmobranch fish (*Scyliorhinus canicula*). J Exp Biol 118: 99–109.

[6] Kalmijn A (1974) The detection of electric fields from inanimate and animate sources other than electric organs. In: Fessard A, editor. Handbook of Sensory Physiology. Berlin: Springer Verlag. pp. 147–200.

[7] Paulin M (1995) Electroreception and the compass sense of sharks. J Theor Biol 174: 325–339.

[8] Montgomery J, Walker M (2001) Orientation and navigation in elasmobranchs: which way forward? Environ Biol Fishes 60: 109–116.

[9] Tricas TC (1982) Bioelectric-mediated predation by swell sharks, *Cephaloscyllium ventriosum* . Copeia 1982: 948–952.

[10] Rivera-Vicente AC, Sewell J, Tricas TC (2011) Electrosensitive spatial vectors in elasmobranch fishes: Implications for source localization. PLoS ONE 6: e16008. 21249147 10.1371/journal.pone.0016008 PMC3020962

[11] Harahush BK, Fischer ABP, Collin SP (2007) Captive breeding and embryonic development of *Chiloscyllium punctatum* Muller & Henle, 1838 (Elasmobranchii: Hemiscyllidae). J Fish Biol 71: 1007–1022.

[12] Cox DL, Koob TJ (1993) Predation on elasmobranch eggs. Environ Biol Fishes 38: 117–125.

[13] Sisneros JA, Tricas TC (2002) Neuroethology and life history adaptations of the elasmobranch electric sense. J Physiology-Paris 96: 379–389. 10.1016/S0928-4257(03)00016-0 14692486

[14] Ballard WW, Mellinger J, Lechenault HA (1993) A series of normal stages for development of *Scyliorhinus canicula*, the lesser spotted dogfish (Chondrichthyes: Scyliorhinidae). J Exp Zool 267: 318–336.

[15] Freitas R, Zhang GJ, Albert JS, Evans DH, Cohn MJ (2006) Developmental origin of shark electrosensory organs. Evol Dev 8: 74–80. 16409384 10.1111/j.1525-142X.2006.05076.x

[16] Moen AN, DellaFera M, Hiller A, Buxton B (1978) Heart rates of white-tailed deer fawns in response to recorded wolf howls. Can J Zoolog 56: 1207–1210. 10.1139/z78-165 667757

[17] Smith EN, Johnson C, Artin KJ (1981) Fear bradycardia in captive eastern chipmunk, *Tamias striatus* . Comp Biochem Phys A 70: 529–532.

[18] Espmark Y, Langvatn R (1985) Development and habituation of cardiac and behavioral responses in young red deer calves (*Cervus elaphus*) Exposed to alarm stimuli. J Mammal 66: 702–711.

[19] Honma A, Oku S, Nishida T (2006) Adaptive significance of death feigning posture as a specialized inducible defence against gape-limited predators. P Roy Soc B 273: 1631–1636. 10.1098/rspb.2006.3501 PMC1634928 16769634

[20] Smith EN (2006) Passive fear: Alternative to fight or flight: When frightened animals hide. New England: iUniverse. pp. 112.

[21] Bowers K, Natterson-Horowitz B (2012) Zoobiquity: What animals can teach us about health and the science of healing. New York: Knopf. pp. 320.

[22] Sisneros JA, Tricas TC (2002) Ontogenetic changes in the response properties of the peripheral electrosensory system in the Atlantic stingray (*Dasyatis sabina*). Brain Behav Evolut 59: 130–140. 10.1159/000064160 12119532

[23] Kalmijn A (1988) Detection of weak electric fields. In: Atema J, Fay RR, Popper AN, Tavolga WN, editors. Sensory biology of aquatic animals. New York: Springer-Verlag. pp. 151–186.

[24] Huveneers C, Rogers PJ, Semmens J, Beckmann C, Kock AA, et al (2012) Effects of the Shark Shield™ electric deterrent on the behaviour of White Sharks (*Carcharodon carcharias*). SARDI Res Rep Ser No. 632.

[25] Kajiura SM, Holland K (2002) Electroreception in juvenile scalloped hammerhead and sandbar sharks. J Exp Biol 205: 3609. 12409487 10.1242/jeb.205.23.3609

[26] Wueringer BE, Squire L, Kajiura SM, Tibbetts IR, Hart NS, et al (2012) Electric field detection in sawfish and shovelnose rays. PLoS ONE 7: e41605. 22848543 10.1371/journal.pone.0041605 PMC3404968

[27] Kalmijn A (1982) Electric and magnetic field detection in elasmobranch fishes. Science 218: 916. 7134985 10.1126/science.7134985

[28] Kajiura SM, Fitzgerald TP (2009) Response of juvenile scalloped hammerhead sharks to electric stimuli. Zoology 112: 241–250. 19097876 10.1016/j.zool.2008.07.001

[29] Fitzgerald TP (2002) Behavioural responses of juvenile sandbar sharks, *Carcharhinus plumbeus* to direct current and alternating current stimuli. Thesis, University of Hawaii.

